# Effects of allyl isothiocyanate fumigation on medicinal plant root knot disease control, plant survival, and the soil bacterial community

**DOI:** 10.1186/s12866-023-02992-w

**Published:** 2023-09-30

**Authors:** Yingbin Li, Daqing Lu, Yan Xia, Xinjing Xu, Huichuan Huang, Xinyue Mei, Min Yang, Jianqiang Li, Shusheng Zhu, Yixiang Liu, Zhiping Zhang

**Affiliations:** 1 Department of Pesticide Science, College of Plant Protection, Yunnan Agricultural University, Kunming, Yunnan, 650201 China; 2 Department of Horticulture, College of Landscape and Horticulture, Yunnan Agricultural University, Kunming, Yunnan, 650201 China; 3 Key Laboratory for Agro-biodiversity and Pest Control of Ministry of Education, Yunnan Agricultural University, Kunming, Yunnan, 650201 China; 4 Department of Plant Pathology, Beijing Key Laboratory of Seed Disease Testing and Control, China Agricultural University, Beijing, 100193 China

**Keywords:** Allyl isothiocyanate, Medicinal plant, Consecutively cultivated soil, Microbial diversity, Soil chemical property

## Abstract

**Background:**

Allyl isothiocyanate (AITC) is a natural product with high volatility that is used as a biofumigant to alleviate soil-borne plant diseases, and problems such as root knot nematodes (RKNs) that necessitate continuous cropping. However, little research has assessed the effects of AITC fumigation on medicinal plants.

**Results:**

AITC significantly reduced the population of RKNs in soil (*p* < 0.0001) and showed an excellent RKN disease control effect within 6 months after sowing *Panax notoginseng* (*p* < 0.0001). The seedling survival rate of 2-year-old *P*. *notoginseng* was approximately 1.7-fold higher after soil treatment with AITC (*p* = 0.1008). 16S rRNA sequencing indicated that the AITC treatment affected bacterial richness rather than diversity in consecutively cultivated (CC) soil. Furthermore, biomarkers with statistical differences between AITC-treated and untreated CC soil showed that Pirellulales (order), Pirellulaceae (family), Pseudomonadaceae (family), and *Pseudomonas* (genus) played important roles in the AITC-treated group. In addition, the microbiome functional phenotypes predicted using the BugBase tool suggested that AITC treatment is more conducive to improving CC soil through changes in the bacterial community structure. Crucially, our research also suggested that AITC soil treatment significantly increases soil organic matter (*p* = 0.0055), total nitrogen (*p* = 0.0054), and available potassium (*p* = 0.0373), which promotes the survival of a succeeding medicinal plant (*Polygonatum kingianum*).

**Conclusion:**

AITC is an ecologically friendly soil treatment that affects the top 10 bacterial richness but not diversity. It could also provide a basis for a useful agricultural soil management measure to alleviate soil sickness.

**Supplementary Information:**

The online version contains supplementary material available at 10.1186/s12866-023-02992-w.

## Background

Medicinal plants have important pharmacological activities and are valued as functional products that can be used as raw materials in the pharmaceutical and food industries. Because of increasing consumption and an associated shortage of raw materials, commercial cultivation of medicinal plants has been implemented in China since 2000 [1]. As an example, *Panax notoginseng* (also known as sanqi), for which over 20,000 ha have been dedicated for cultivation and the annual production in recent years has exceeded 20 million kg, provides ingredients for more than 2,000 products [2, 3]. However, continuous cropping of medicinal plants in the field is limited by soil sickness, which is a form of negative plant–soil feedback that reduces crop yield and occurs when the same crop or a related species is cultivated successively in the same soil [4]. High seedling death rates due to root rot and root-knot nematode disease in many commercial medicinal plant production areas seriously affect plant growth and yield [3, 5]. These diseases also cause major changes in the physicochemical and biological properties of soil [6, 7]. Soil sickness is caused by a combination of biotic and abiotic factors that disturb the biological balance of the soil [8], including accumulation of soil-borne pathogens, autotoxicity, soil microbial community imbalance, deterioration of soil physicochemical properties, unbalanced soil nutrients, and environmental stresses [3, 4, 9].

Soil sterilization using compounds such as dazomet, dimethyl disulfide, metam-sodium, methyl-bromide, and chloropicrin is an effective and common treatment for controlling soil-borne pests and diseases [10–12]. However, the use of most soil fumigants is forbidden or strictly restricted for resistance-related, environmental, and safety reasons [13, 14]. Pesticide residues have significantly reduced the quality of medicinal plants, thereby affecting human health [15, 16]. Therefore, there is an urgent need to identify a safe and effective soil fumigant to overcome soil sickness during medicinal plant cultivation.

Allyl isothiocyanate (AITC), which is derived from plant material in an ecologically friendly manner, has been used in agriculture [17] because of its fungicidal oomyceticidal, bactericidal [18], nematocidal [19], and herbicidal [20] biological activities. The United States Environmental Protection Agency has licensed AITC for use as a biological pesticide [21]. The proposed action mechanisms of AITC include inducing glutathione S-transferase (GST) expression in *Caenorhabditis elegans* [22], affecting protein structures by disrupting disulfide bonds in bacteria [23], and killing fungal cells by eliciting an oxidative stress response as in the case of *Fusarium solani* for soil-borne disease control [24]. In addition, isothiocyanates (ITCs) can induce suicidal germination in some plant seeds [25], and AITC fumigation of first-generation tomato soil promoted second-generation tomato plant growth and had a “fertilizer effect” [26]. However, ITCs can inhibit the germination of peas, wheat, and rapeseed was observed by reducing respiration and anaerobic glycolysis [27], and significantly reduced *Cyperus rotundus* densities in a drip fumigation experiment [28], suggesting that different hosts respond to ITCs with different levels of sensitivity.

Treatment of soil with synthetic fumigants often has a significant impact on soil microbial communities. Chloropicrin greatly reduced soil biomass and bacterial species richness, influenced bacterial community structure, and affected non-target microorganisms [29, 30]. In addition, bacterial community diversity associated with biodegradation increased significantly, and denitrification was significantly promoted, suggesting negative effects on the environment [31]. Methyl bromide soil treatment resulted in a shift toward a community dominated by Gram-positive bacterial biomass [32]. However, some pesticides have suppressive or no effects on microorganisms. For example, diuron and chlorotoluron showed no differences between treated and non-treated soil, while linuron had a marked difference [33]. Biofumigated mustard greens (*Brassica juncea)* caused much less damage to the soil bacterial community than chemical chloropicrin [29]. Biofumigation with rapeseed (*Brassica napus* ‘Dwarf Essex’) meal increased bacterial diversity but decreased fungal diversity [34]. Zhu et al. [35] found that AITC fumigation has a relatively small effect on the soil bacterial community, but significantly changed the structure of the soil fungal community in tomato production. Low-dose AITC had no significant effect on soil bacterial richness, and only temporarily inhibited the diversity of bacterial phyla, while high-dose AITC inhibited the diversity and richness of the bacterial community over longer periods. Other studies found that the richness of Proteobacteria and Firmicutes bacteria increased significantly in the short term after pesticide soil treatment, while the richness of sulfur bacteria decreased significantly in the short term, which may be related to its strong stress resistance [29, 36].

Although AITC can control soil-borne diseases effectively, the effects on soil sickness in the context of medicinal plants require further evaluation. Thus, this study aimed to determinate the effects of AITC soil treatment on the survival of medicinal plants (*Panax notoginseng* and *Polygonatum kingianum*) in a consecutively cultivated (CC) soil system. In addition, the response of the soil bacterial community and changes of soil chemical properties associated with AITC fumigation were clarified.

## Methods

### Chemicals

Commercial 20% AITC EW and 0.5% avermectin GR were purchased from Jiangsu Teng-Long Biological Pharmaceutical and Guangdong Zhenge Biotechnology, respectively.

### Plants and their characteristics

*Panax notoginseng* seeds were purchased from Wenshan Sanqi Trading Market (Geo-Authentic Product Area), and were sown during November–December in a nursery to grow 1-year-old seedling. In a field test, we also transplanted the roots of 1-year-old seedlings measuring 9–12 cm during November–December for 2-year-old growth [3]. *Polygonatum kingianum* seeds were purchased from Lancang Country, and sown in a nursery in January. *P. notoginseng* and *P*. *kingianum* both demand shade, and are important herbal medicines in China.

### Pot experiment

A pot experiment was conducted from December 2021 to July 2022 at the Agricultural Experimental Station of Yunnan Agricultural University, Xundian County, Kunming, China (25.521° N 103.286° E; altitude of 1,960 m). The test soil was collected from a *P. notoginseng* seedling base in Lancang County, where nematode disease was serious. Healthy seeds were sown in bowls (0.05 m × 0.05 m spacing, 10 seeds per bowl) with a soil thickness of 0.15 m (the height from the bottom of the bowl to the soil surface) in December 2021. Each treatment had 10 replicates. Before sowing, 20% AITC EW was applied at doses of 0, 15, 45, or 75 L/ha for soil treatment by an irrigation method; untreated soil (CK), soil treated by steaming at 80 °C for 30 min, and soil treated with avermectin at a dose of 45 kg/ha were used as controls, respectively. The seedling survival rate (SSR) was recorded and the occurrence of root knot disease was investigated in July 2022 using previously described methods [37, 38].

### AITC soil treatment method for the pot experiment and greenhouse assessment

First, 20% AITC EW was mixed with water to create a series of final concentrations, and 30 L/m^2^ irrigation volume was provided to ensure that the AITC could fully penetrate into the soil. Second, black polyethylene film (thickness 0.005–0.01 mm) was applied as a soil wrapping to create a tight seal. After 7 days of fumigation, the black polyethylene film was removed. Another 7 days of sun-curing was then necessary for the subsequent sowing or transplanting operations.

### Evaluating the effect of AITC soil treatment on the growth of medicinal plants in a greenhouse

The effects of AITC treatment on the growth of 2-year-old sanqi were also assessed in the Agricultural Experimental Station of Yunnan Agricultural University, Xundian County, Kunming, China, where sanqi plants have been cultivated continuously from 2015 to 2021. The characteristics of the CC soil were as follows: pH, 7.49; electrical conductivity, 151.1 µs/cm; available phosphorus, 86.18 mg/kg; available potassium, 809.56 mg/kg; and organic matter (OM), 36.78 g/kg. Before seedling transplantation, 20% AITC EW was applied at a dose of 45 L/ha for CC soil treatment.

Healthy *P. notoginseng* seedlings were transplanted into AITC-treated CC soil with 0.10 m × 0.10 m spacing. Each plot had an area of 5 m^2^ (about 500 plants) and five replicates were used for each treatment using a randomized block design. CC soil without AITC treatment was also transplanted as a control. To mimic the natural conditions for sanqi growth, the greenhouse was shaded with a polyethylene net that allowed 10% light transmission. The temperature was controlled at 18–30 °C and strict moisture control was also implemented. The seedling emergence rate (SER) was recorded when the plant emergence rate in CC soil exceeded 50% (1 June). The SSR was recorded 2 months later (1 August). The “seedling survival fold value” was calculated as follows: fold value = (number of seedlings in treatment group − number of seedlings in control group) / (number of seedlings in control group).

To evaluate the effect of AITC soil treatment on the growth of succeeding crops in CC soil, another traditional Chinese medicinal plant, *Polygonatum kingianum*, was sown (0.05 m × 0.05 m spacing) in January 2022 after 2-year-old sanqi were harvested (at 1-month intervals). Briefly, seeds were coated with fludioxonil, and a 2,000 m^2^ area of soil was selected for AITC disinfectant treatment at a dose of 45 L/ha. AITC untreated CC soil was sown as a control. Three replicate plots (each with an area of 6 m^2^) were used. The greenhouse environmental conditions were consistent with those described above. The SSR per square meter was recorded and analyzed on June 17 and September 2, respectively.

### Soil sampling

The CC soil was treated with AITC at 45 L/ha according to the method described above, and soil samples were collected before seedling transplantation and untreated CC soil samples were collected as the control. Briefly, the soil samples were collected randomly from 20 pots and mixed into three biological replicates for each treatment. All samples were placed in 5 mL centrifuge tubes and stored at − 80 °C for DNA extraction and high-throughput sequencing.

### DNA extraction and high-throughput sequencing

The total genomic DNA of each soil sample (200 mg) was extracted using the Fast DNA Spin Kit for soil (MP Biomedicals, Solon, OH, USA) according to the manufacturer’s instructions. The full length of the bacterial 16 S rRNA gene was amplified using the primer sets 27 F (5ʹ-AGRGTTTGATYNTGGCTCAG-3ʹ) and 1492R (5ʹ-TASGGHTACCTTGTTASGACTT-3ʹ), and followed thermal conditions: initial denaturation at 95 °C for 3 min, followed by 27 cycles at 95 °C for 30 s, 55 °C for 30 s, 72 °C for 45 s, and 72 °C for 10 min. The purified amplicons were sequenced using the single molecule real-time sequencing (SMRT) method and a PacBio Sequel sequencing platform (PacBio Menlo Park, CA, USA) according to the standard protocol. The aforementioned operations were completed by Biomarker Technologies Corporation (Beijing, China).

### Bioinformatics

First, the raw circular consensus sequences (CCS) were identified and generated based on barcode sequences using lima v1.7.0 (default parameters) (https://github.com/pacificbiosciences/barcoding). Then, the primer sequences were identified and removed using cutadapt v2.7 (maximum allowable primer mismatching rate of 20%) [39], and the raw CCS sequences were filtered to generate clean CCS sequences (1,200–1,650 bp). The clean sequences were clustered into operational taxonomic units (OTUs) at the 97% similarity using USEARCH (ver. 10.0) [40]. Taxonomic annotation of feature sequences was first processed with the Naive Bayes Classifier(through the “classify-sklearn” method in QIIME2), and then blasted using SILVA (Release132, http://www.arb-silva.de) to determine the species composition of soil communities [41].

### Soil chemical property analyses

The AITC-treated and untreated CC soils described above were sieved (2 mm diameter) and oven dried. The soil pH was determined using PHS-3E (Leici; Shanghai, China) in a 1:2.5 soil/water (w/v) suspension according to standard NY/T 1121.2–2006. Soil OM was assayed using dichromate wet combustion according to standard NY/T 1121.6–2006. Soil total nitrogen (TN) content was analyzed using an azotometer (SKD-1000, Kjeldahl, Shanghai, China) according to standard NY/T 53-1987. Soil available phosphorus (SAP) was analyzed using an ultraviolet–visible spectrophotometer (T6 series; PERSEE, Auburn, CA, USA) according to standard NY/T 1121.7–2014, and soil available potassium was analyzed by flame atomic absorption spectrophotometry (Z-2310; Hitachi, Tokyo, Japan) according to standard NY/T 1121.7–2014. All soil samples from each treatment were tested with three replications.

### Statistical analysis

Seedling survival, the alpha diversity index, the number of species types, and soil chemical properties were analyzed using GraphPad Prism 8.3 software (GraphPad Software, La Jolla, CA, USA), which was also used for data visualization. The microbial taxa data were analyzed using R ver. 2.15.3 (R Foundation for Statistical Computing, Vienna, Austria). Statistics on the compositions of each sample were calculated at the phylum, class, order, family, genus, and species levels. Alpha diversity indices (Chao1, ACE, Shannon, and Simpson) and beta diversity were calculated using QIIME software (https://qiime2.org/) [42]. To compare bacterial community structure between the AITC-treated and untreated samples, principal coordinates analysis (PCoA) was performed based on the Bray–Curtis distance at the OTU level, in which the horizontal (PC1 axis) and vertical (PC2 axis) coordinates were the main principal components contributing to the differences in soil bacterial community composition among all samples. The samples were clustered hierarchically using the unweighted pair group method with arithmetic mean (UPGMA). The linear discriminant analysis (LDA) effect size (LEfSe) was used to identify statistically different biomarkers among groups (the default LDA score is 4.0). Microbiome functional phenotypes were predicted using the BugBase tool. The data were obtained from BMK Cloud (www.biocloud.net). Two-way analysis of variance (ANOVA) and the unpaired *t*-test were used for statistical analysis. All illustrations were created using Adobe Photoshop CS6 software (Adobe, Mountain View, CA, USA).

## Results

### Effects of AITC soil treatment on 1-year-old seedling survival and the occurrence of root knot disease in the pot experiment

**Fig. 1:**
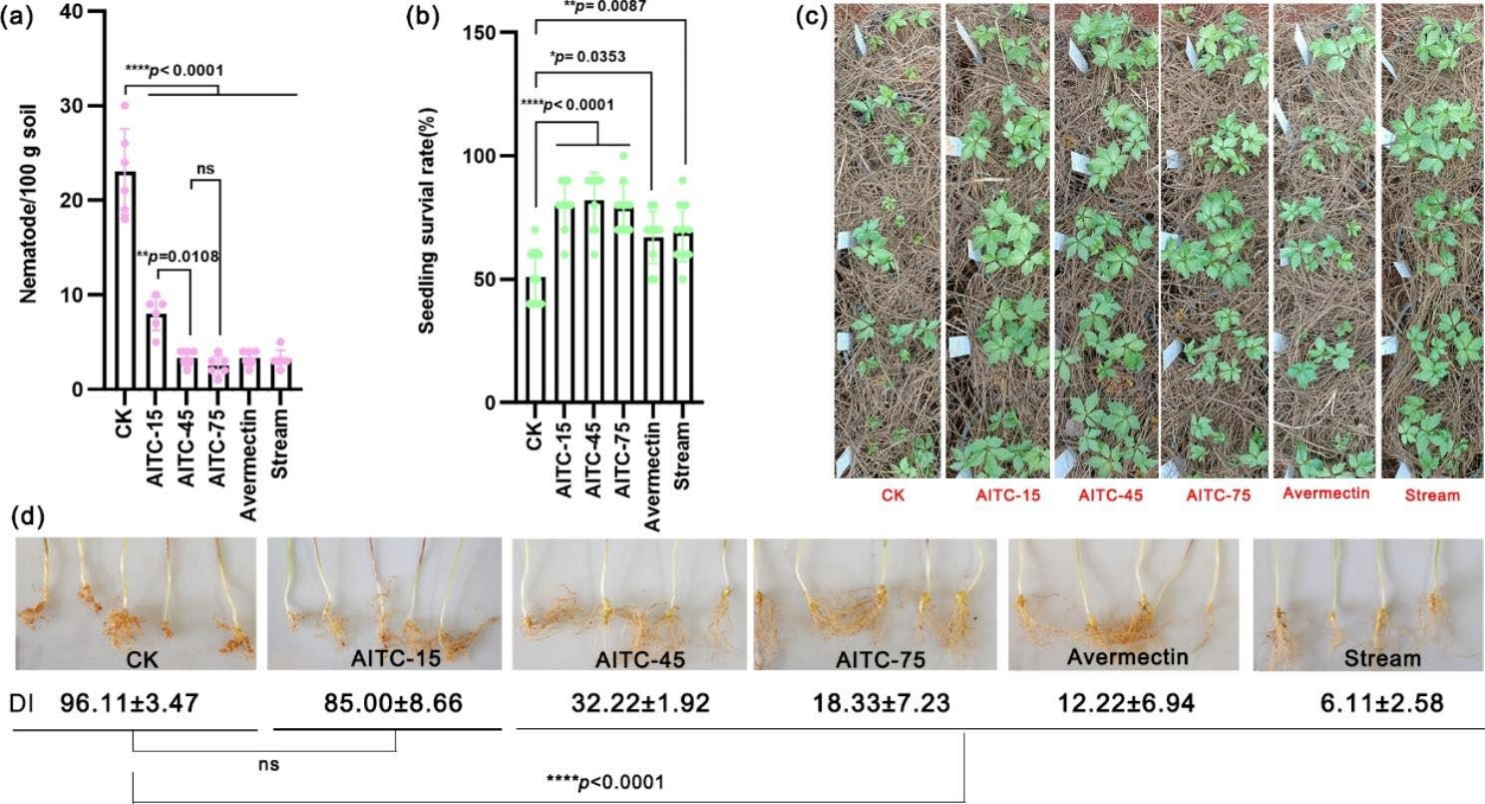
Seedling survival of 1-year-old *P*. *notoginseng*. **(a)** The number of nematodes (*n* = 6). **(b)** Seedling survival rate in July 2022 (*n* = 10). **(c)** Seedling growth in plot in July 2022. **(d)** Occurrence of root knot nematode disease (*n* = 3). Untreated soil (CK), avermectin treatment, and steam treatment were used as controls. AITC-“*X*” indicates soil treatment with AITC at a dose of 15, 45, or 75 L/ha. “DI”, means disease index; “ns”, no significant difference. All data are presented as mean ± standard deviation (SD) and were compared using two-way ANOVA.

As shown in Fig. 1a, the number of nematodes was significantly lower in the soil treated by the different methods (*p* < 0.0001) than in the untreated CK soil (23.00 ± 4.56). AITC treatment at doses of 15, 45, and 75 L/ha decreased the number of nematodes in the soil to 8.00 ± 1.79, 3.33 ± 0.82, and 2.50 ± 1.05 per 100 g soil, respectively. The 45 and 75 L/ha treatments were better than the 15 L/ha treatment (*p =* 0.0108). Avermectin and steam treatment also decreased the number of nematodes, to 3.33 ± 0.82 and 3.17 ± 0.98, respectively. The SSR for CK was about 51%, whereas the AITC treatment groups achieved the highest SSR (> 80%; *p* < 0.0001). Avermectin and steam treatment also maintained a high SSR of approximately 70% (Fig. 1b and c). Notably, AITC soil treatment at rates of 45 and 75 L/ha showed high ability to control root knot disease within 6 months after sowing (*p* < 0.0001) (Fig. 1d).

### Effect of AITC soil treatment on 2-year-old sanqi survival in the greenhouse

**Fig. 2:**
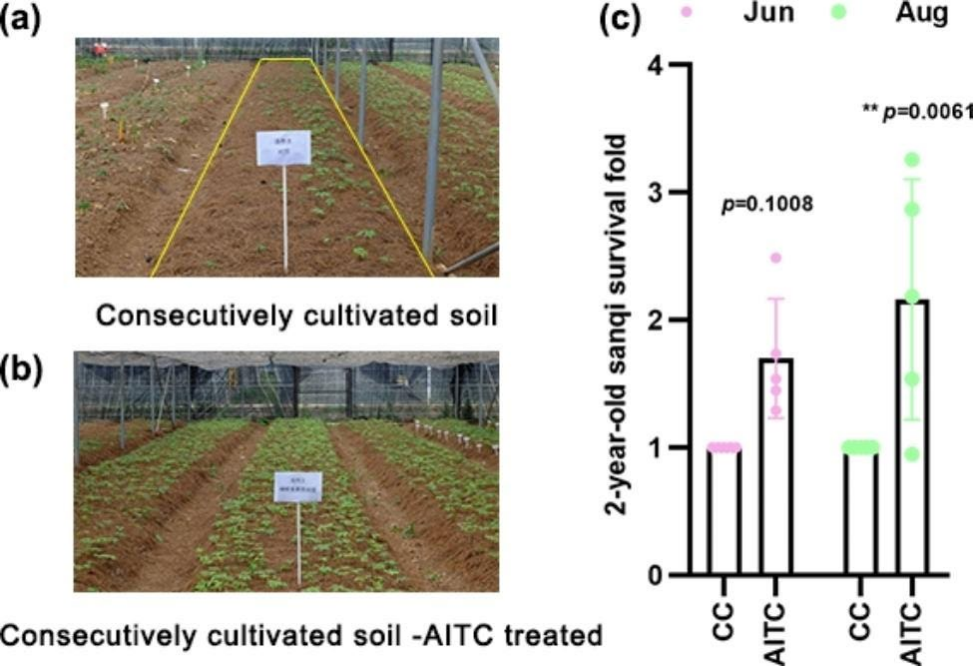
Survival of 2-year-old *P*. *notoginseng*. **(a)** Consecutively cultivated (CC) soil (the area marked with the yellow line). **(b)** AITC-treated CC soil. **(c)** Survival fold values in June and August. *n* = 5 for the CC/AITC treatment group. All data are presented as mean ± standard deviation (SD) and were compared using two-way ANOVA.

To further evaluate the effect of AITC on the growth of 2-year-old *P. notoginseng* further, a greenhouse experiment was conducted. Compared to plants in untreated CC soil, AITC soil treatment promoted 2-year-old sanqi survival (Fig. 2a and b). As shown in Fig. 2c, the SSR in June was approximately 1.7-fold higher in soil treated with AITC (*p* = 0.1008) than in CC soil. Two months later, 2-year-old sanqi in CC soil died rapidly due to root disease. However, the SSR was significantly higher when CC soil was treated with AITC at 45 L/ha. It was approximately 2.16-fold higher compared to CC soil in August (*p* = 0.0061).

### Effect of AITC treatment on the survival of succeeding crops in CC soil

**Fig. 3:**
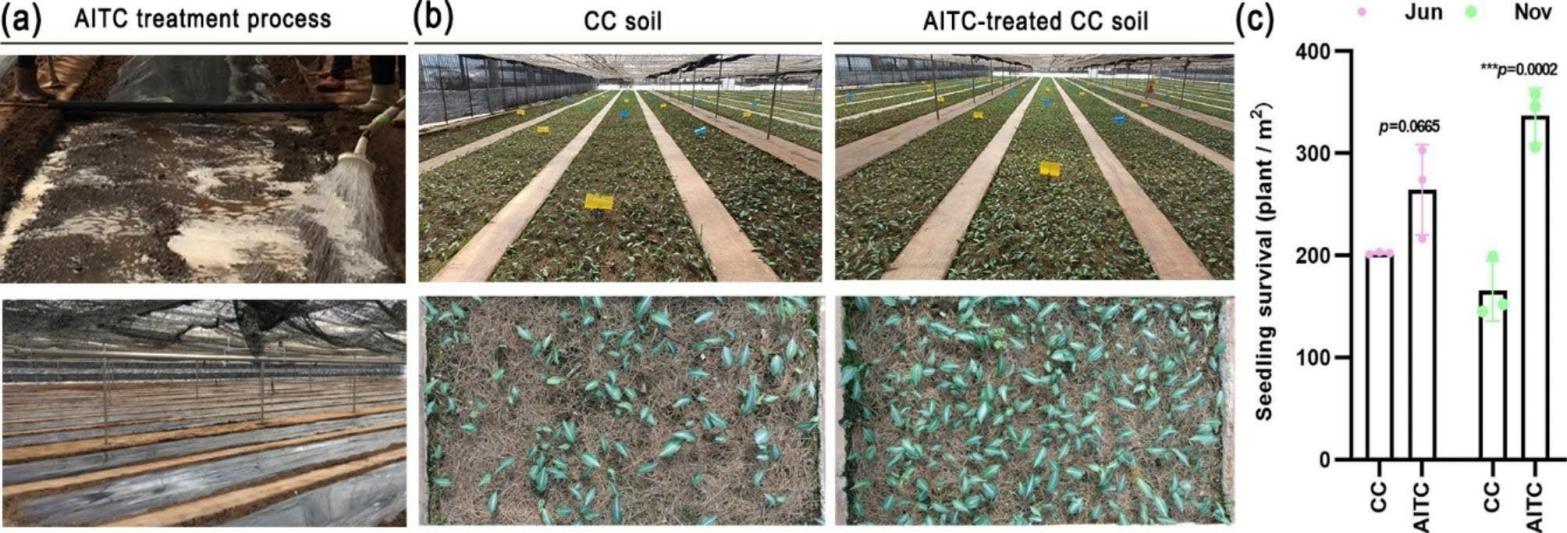
Seedling survival of *P*. *kingianum* in CC soil. **(a)** AITC treatment process. **(b)** Seedling growth in CC and AITC-treated CC soil. **(c)** Seedling survival in June and November, respectively

To evaluate the effect of AITC on succeeding crop growth after the harvest of *P*. *notoginseng* in more detail, another traditional Chinese medicinal plant, *P*. *kingianum*, was sown after AITC treatment in CC soil (Fig. 3a). As shown in Fig. 3b, seedling survival was significantly better in the AITC-treated than untreated CC soil. The SSR was approximately 1.3-fold higher after soil treatment with AITC (*p* = 0.0665) compared with CC soil (about 202 plants per square meter) in June. Five months later, the seedling survival rate of *P*. *kingianum* in CC soil had gradually diminished. However, the rate was significantly higher when CC soil was treated with AITC at a concentration of 45 L/ha. The SSR was about 2.0-fold higher compared with CC soil (about 337 plants per square meter) in November (*p* = 0.0002) (Fig. 3c).

### Effects of AITC on the alpha diversity of soil bacteria

**Fig. 4:**
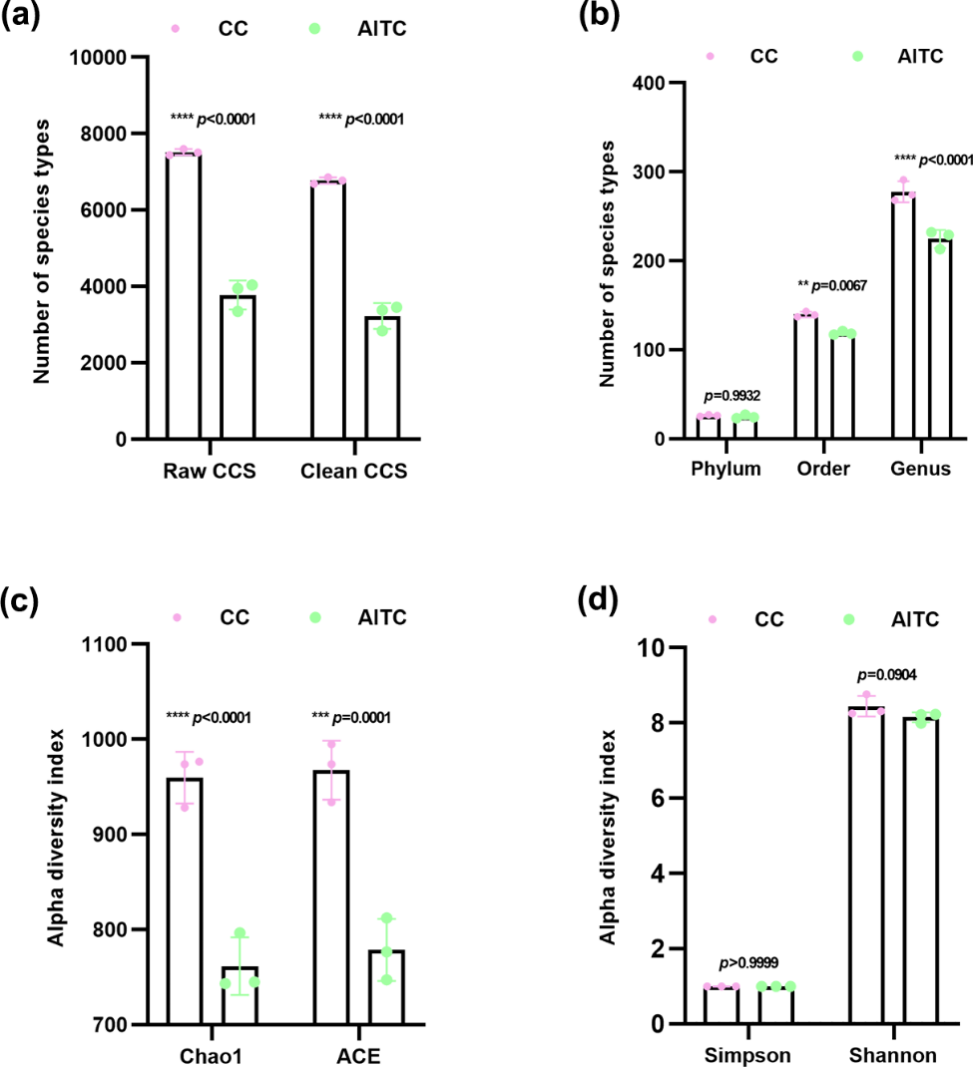
Alpha diversity indices of bacterial communities with and without treatment with AITC. **(a)** Raw CCS data: the number of CC sequences in each sample; Clean CCS data: the number of sequences after primer removal and length filtering (< 0.005%). **(b)** Number of species types at each level (phylum, order, and genus) of the soil bacterial community. (**c** and **d**) Alpha diversity analysis including the Chao1, ACE, Simpson, and Shannon indices. *n* = 3 for CC/AITC treatment group. All data are presented as mean ± standard deviation (SD) and were compared using two-way ANOVA.

As in Fig. 4 shows, there were significantly fewer circular consensus sequences in both the raw and clean CCS data (*p* < 0.0001) after AITC soil treatment at 45 L/ha than in CC soil, suggesting that AITC readily kills bacteria and causes DNA degradation in soil (Fig. 4a). Interestingly, AITC application did not affect soil bacterial richness at the phylum level (*p* = 0.9932) but did decrease richness at the order (*p* = 0.0067) and genus (*p* < 0.0001) levels (Fig. 4b). The alpha diversity indices, such as Chao1 and ACE, were significantly lower after CC soil was treated with AITC (Fig. 4c), while there were no statistical differences in the Simpson (*p* > 0.9999) and Shannon (*p* = 0.0904) indices between the CC and AITC-treated soil (Fig. 4d). Combined, these results indicate that AITC treatment affected bacterial richness rather than diversity in CC soil.

### Biomarkers with significant differences between treated and untreated samples

**Fig. 5:**
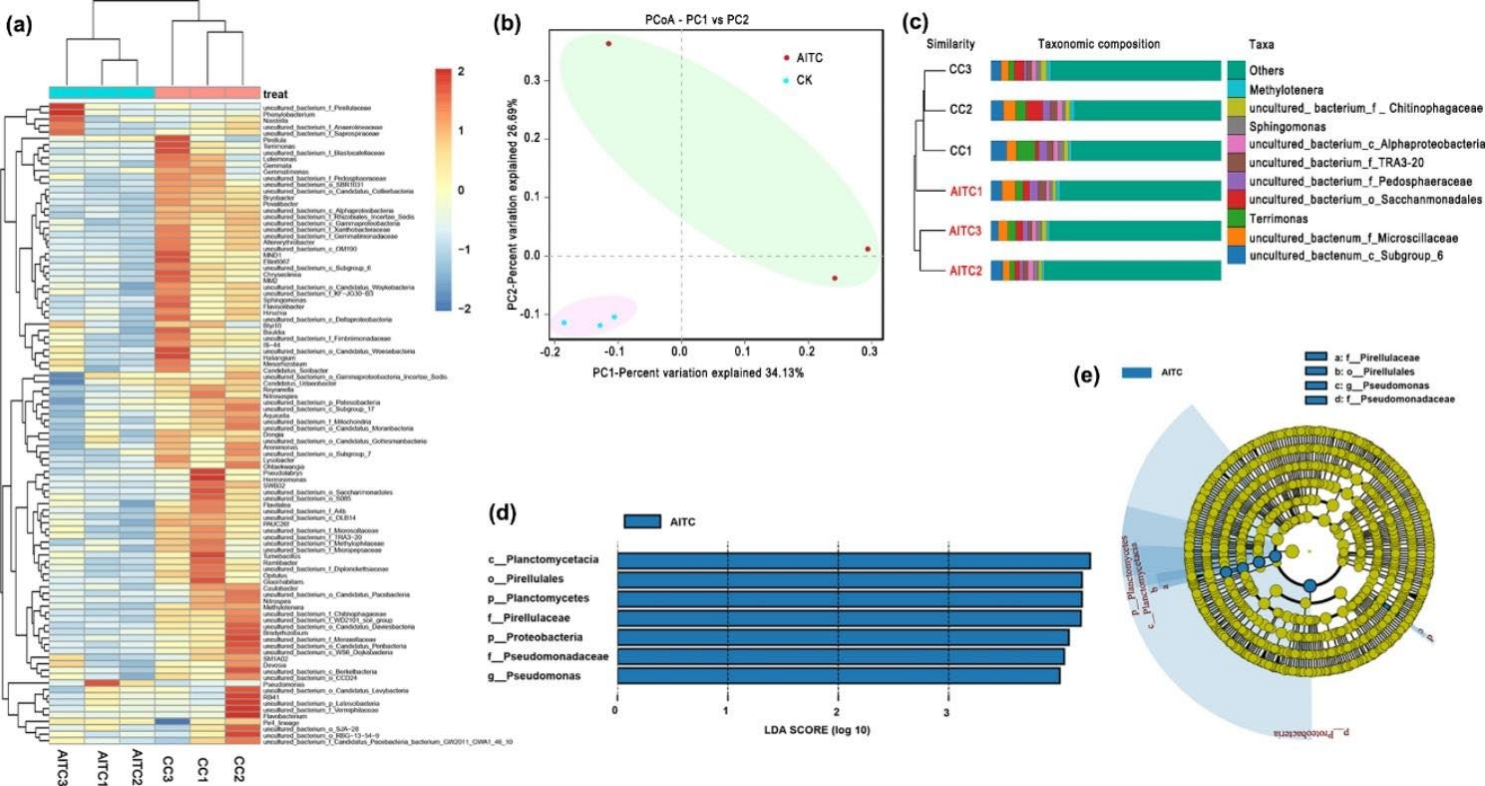
The bacterial composition heatmap, beta diversity and LEfSe analyses. **(a)**. Taxonomic cluster heatmap generated by z-normalization of relative species abundances. **(b)** Principal coordinates analysis (PCoA) based on Bray–Curtis dissimilarity index of bacterial communities with and without treatment with AITC. **(c)** Unweighted pair group method with arithmetic mean (UPGMA) clustering of the bacterial communities associated with all soil samples, the rest of the top 10 genus were combined as “others”, “unclassified” represents the species that has not been taxonomically annotated. **(d)** Species with a linear discriminant analysis (LDA) score greater than the set value (default setting is 4.0). The lengths of the histograms represent the impact of different species. **(e)** Phylogenetic dendrogram of bacterial biomarkers among all soil samples. *n* = 3 for the CC/AITC treatment group

The bacterial composition heatmap showed that the soil bacteria in CC soil (CC1, CC2, and CC3) and CC soil treated with AITC (AITC1, AITC2, and AITC3) were divided into two clusters. Most of the bacteria units had lower relative abundances (*p* < 0.05), with standardization *Z* values ranging from − 2 to 0, in the AITC group than in CC soil (*Z* value range of 0 to 2) (Fig. 5a, Supplementary Table S1); however, some bacterial genera had higher relative abundances (Fig. 5a, Supplementary Table S2) (*p* < 0.05).

The total variance explained in the bacterial community composition of the samples was 60.82% (PC1, 34.13%; PC2, 26.69%) (Fig. 5b), indicating that AITC treatment was the major factor contributing to the differences in bacterial community composition.

The top 10 bacterial taxa in the community structure were analyzed further; the UPGMA was calculated through analysis of high-throughput sequencing results. As shown in Fig. 5c, the dominant bacterial genera were *uncultured_bacterium_c_Subgroup_6*, *uncultured_bacterium_f_Microscillaceae*, *Terrimonas*, *uncultured_bacterium_o_Sacchanmonadales*, *uncultured_bacterium_f_Pedosphaeraceae*, *uncultured_bacterium_f_TRA3-20*, *uncultured_bacterium_c_Alphaproteobacteria*, *Sphingomonas*, *uncultured_ bacterium_f _ Chitinophagaceae*, and *Methylotenera*. Notably, although AITC-treated and untreated bacteria were obviously divided into two clusters, the bacterial community compositions of each cluster were not significantly different (*p* > 0.05), no matter the level (phylum, class, order, family, or genus) (Fig. 5c and S1). This shows that AITC treatment did not affect the top 10 bacterial composition in CC soil.

Biomarkers with statistical differences between AITC-treated and untreated samples were subjected to LDA. As shown in Fig. 5d, the bacterial sequences were predominantly associated with Planctomycetacia (class), Planctomycetes (phylum), Pirellulales (order), Pirellulaceae (family), Proteobacteria (phylum), Pseudomonadaceae (phylum), and *Pseudomonas* (genus). Among these bacterial taxa, Pirellulales (order), Pirellulaceae (family), Pseudomonadaceae (family), and *Pseudomonas* (genus) played important roles in the AITC-treated group (Fig. 5e).

### BugBase predictions of microbiome phenotypes in treated and untreated samples

**Fig. 6:**
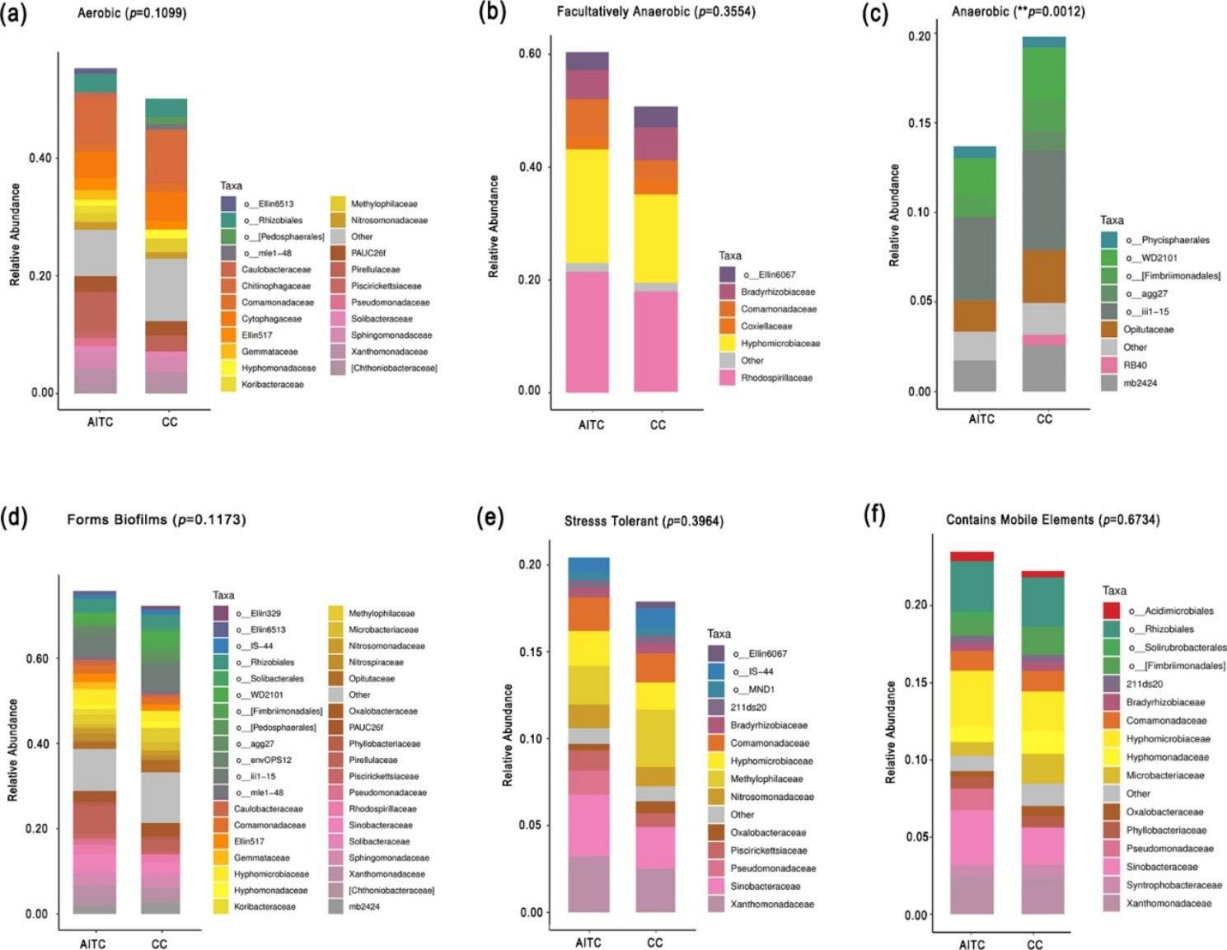
BugBase prediction of microbiome phenotypes in treated and untreated samples. **(a)** Aerobic. **(b)** Facultatively anaerobic. **(c)** Anaerobic. **(d)** Biofilm-forming. **(e)** Stress tolerant. **(f)** Mobile element-containing. *n* = 3 for the CC/AITC treatment group. All data are presented as means and were compared using the unpaired *t* test

The microbiome functional phenotypes in the treated and untreated samples, including aerobic, facultative anaerobic, anaerobic, biofilm-forming, oxidative stress tolerant, and mobile element-containing phenotypes, were predicted using the BugBase tool according to their richness (Supplementary Table S3). Compared with CC, the richnesses of aerobic (Fig. 6a), facultative anaerobic (Fig. 6b), biofilm-forming (Fig. 6d), oxidative stress tolerant (Fig. 6e), and mobile element-containing bacteria (Fig. 6f) trended to increase, albeit without significant differences (*p* > 0.05), while that of anaerobic bacteria (Fig. 6c) decreased significantly (*p* = 0.0012), after AITC treatment in CC soil.

### Effect of AITC treatment on soil chemical properties

**Fig. 7:**
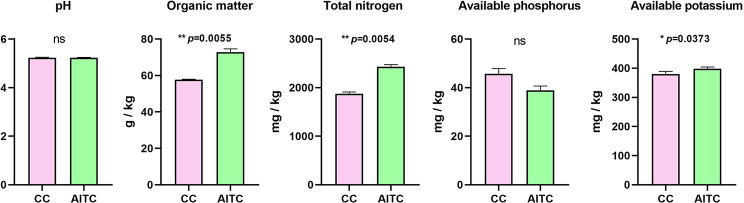
Seedling survival of *P*. *kingianum* and chemical changes in CC soil. **(a)** AITC treatment process. **(b)** Seedling growth in CC and AITC-treated CC soil. **(c)** Seedling survival in June and November, respectively. **(d)** Chemical changes of CC soil 7 days after fumigation with AITC. *n* = 3 for the CC/AITC treatment group. “ns”, no significant difference. All data are presented as mean ± standard deviation (SD) and were compared using two-way ANOVA.

The soil chemical properties of AITC-treated and untreated CC soil were compared (Fig. 7). The AITC soil treatments significantly increased the OM (*p* = 0.0055), TN (*p* = 0.0054), and SAP (*p* = 0.0373) values in comparison with the values in untreated CC soil. In addition, both soil pH and soil available potassium showed no significant difference between treated and untreated CC soil.

## Discussion

The growth of medicinal plants is often restricted by soil diseases, and traditional prevention and control methods using chemical pesticides are not conducive to sustainable development [3]. Soil fumigation is widely applied to increase crop yield and satisfy global food demand [43], and biofumigation is an ecofriendly alternative to chemical fumigation that has been used to control soil-borne plant diseases [19, 34]. In this study, we first evaluated the ability of AITC fumigation to control medicinal root knot disease and promote plant growth. We found that both *P. notoginseng* seedlings and *P. kingianum* seeds showed higher SSR after CC soil was treated with AITC. Importantly, our research revealed, for the first time, the effects of AITC on the soil microbial diversity and community structure of medicinal plants.

### AITC promotes medicinal plant growth by killing nematodes and enriching specific probiotics

AITC is the predominant isothiocyanate obtained from damaged *Brassica* tissues and has been used as a fumigant for controlling soil-borne diseases because of its low impact on the environment and low risk of persistence [24, 44, 45]. In our study, AITC was applied to control soil sickness by reducing the number of nematodes in the soil, changing the composition of functional microorganisms, and promoting medicinal plant survival in a CC system. According to our research, AITC soil treatment improves the SSR of medicinal plants, while significantly reducing the number of nematodes in CC soil; some AITC-tolerant probiotics are significantly enriched (such as *Pseudomonas* etc.), and these probiotics are reported to be fungistatic. AITC modifies the biophysical root environment, induces plant disease resistance [46, 47], and promotes nutrient absorption (such as phosphate solubilization activity, etc.). These findings provide evidence that AITC promotes the growth of medicinal plants.

In pepper soil, AITC soil fumigation decreased the richness of *Planctomycetes*, *Acinetobacter*, *Pseudodeganella*, and RB41, but increased those of *Lysobacter*, *Sphingomonas*, *Pseudomonas*, *Luteimonas*, *Pseudoxanthomonas*, and *Bacillus*, at the genus level [48]. In tomato soil, there were significant increases in the richness of probiotics, such as *Sphingomonas* and *Streptomyces*, following AITC fumigation [35]. However, in medicinal plant soil, *Planctomycetes*, *Acinetobacter*, *Pseudodeganella*, *Sphingomonas*, and *Streptomyces* were not detected by high-throughput sequencing, while *Pseudomonas* and *Pseudoxanthomonas* were significantly enriched. AITC soil fumigation treatment has various effects on different planting crops and one potential reason is that the soil microbiota is shaped by native plants [49].

### AITC is relatively safe for fumigating medicinal plant soil compared to synthetic fumigants

Biofumigant mustard greens (*Brassica juncea*) cause much less disturbance of the soil bacterial community than the chemical chloropicrin, and AITC fumigation had less effect on bacteria than on fungal communities, which reduced the diversity of tomato soil bacteria temporarily [29, 35]. Compared with those studies, our research further revealed that AITC soil fumigation significantly lowered the Chao1 and ACE indices, but did not affect the Simpson and Shannon indices, suggesting that AITC treatment affected bacterial richness rather than diversity in CC soil of medicinal plants. Considering the short half-life of AITC in soil [29, 35], we speculate that the bacterial richness would increase as the application period is lengthened; however, determining this requires further soil sample collection and sequencing analysis at different times after AITC treatment. In combination with previous reports, we believe that AITC is a relatively safe fumigation method for treating medicinal plant soil compared to synthetic fumigants (such as chloropicrin), which significantly decreased the bacterial community diversity, affected soil function, and had negative effects on the environment surrounding fumigated soils [50].

### AITC soil fumigation recruits PGPR and improves soil properties

*Brassica* plants are often sources of isothiocyanates (ITCs), which could affect the soil microbial community during growth. Previous reports have shown that *Brassica* plants are the source of an enormous number of plant growth-promoting rhizobacteria (PGPR) that directly and indirectly promote plant growth. Some of the PGPR frequently isolated from *Brassica* species include *Agrobacterium*, *Pseudomonas*, and *Rhizobium* [43, 51]. *Panax notoginseng* is a perennial and studies have reported a negative relationship between the death rate of *P. notoginseng* and bacterial community dynamics in that the ratio of fungi to bacteria increased significantly with the number of planting years in a CC system [46]. Thus, increasing the bacterial community diversity and richness is an important way to alleviate obstacles to continuous cropping, especially for PGPR with potential antagonistic effects on soil pathogenic fungi, such as *Fusarium* spp. and *Ilyonectria* spp. [3]. Consistent with those findings, many reported beneficial taxa in PGPR, such as *Pseudomonas* and *Pseudoxanthomonas* [52–56], were also found to be more abundant in our study, with the direct use of the metabolite AITC of *Brassica* plants for soil fumigation.

Soil microorganisms exist mainly in biofilms, and the formation of biofilms can help microorganisms gain ecological advantages, such as by gaining resistance to dry environments, changing the soil microenvironment, enhancing the viability of bacteria, and affecting soil chemical properties [57, 58]. In our study, the CC soil became loose and porous after AITC fumigation, which may occur because AITC promotes soil-aggregate restoration [43]. In addition, this phenomenon might lead to an increase in the oxygen content of the soil and explain the significant decrease in anaerobic bacteria and the increasing trends in the richness of aerobic, facultative anaerobic, biofilm-forming, oxidative stress tolerant, and mobile element-containing bacteria.

Soil microbes play a key role in nutrient cycling [59]. In a prior study, the soil OM of mustard (*Brassica* plants)–eggplant treatment was 2.65 times greater than that of continually planted eggplant treatment [50]. OM was also increased after the AITC treatment in our study; TN and soil available potassium also improved significantly, One possible cause is that AITC soil fumigation enriched the numbers of aerobic denitrifying bacteria (inspired by the BugBase prediction that the relative abundance of Pseudomonadaceae increased from 0 to 0.014 after AITC soil fumigation treatment, Supplementary Table S3), which promotes the decomposition of plant residues in the process of growth and reproduction in the soil. However, studies have also shown that *Pseudomonas* and *Pseudoxanthomona* are carbon-fixing and methanogenic microorganisms; they can use hydrogen as energy, synthesizing organic carbon from inorganic carbon [60]. The mechanisms by which AITC increases soil OM, TN, and soil available potassium remain to be elucidated.

## Conclusions

Soil sickness results in plant–soil feedback that reduces crop yield. The ecologically friendly method introduced herein provides new insight into soil-borne disease suppression and promotes medicinal plant growth for the current and subsequent crop. In addition, for the first time our study shows that AITC affects soil microbial richness but not diversity. Importantly, soil probiotic bacteria were significantly enriched in soil after AITC fumigation, which could be of great significance for understanding CC obstacles and providing information for screening beneficial microbes for the management of medicinal plant health.

### Electronic supplementary material

Below is the link to the electronic supplementary material.

Supplementary Material 1

Supplementary Material 2

Supplementary Material 3

Supplementary Material 4

## Data Availability

The original contributions presented in the study are publicly available. These data can be found in the Short Read Archive (SRA) at NCBI database under accession number PRJNA917239 (https://www.ncbi.nlm.nih.gov/bioproject/PRJNA917239). The datasets analyzed during the current study are available from the corresponding author on reasonable request.
