# Supplementary material for: Effects of allyl isothiocyanate fumigation on medicinal plant root knot disease control, plant survival, and the soil bacterial community
Source: BMC Microbiol. 2023 Sep 30;23:278. doi: 10.1186/s12866-023-02992-w (PMC10542678; doi:10.1186/s12866-023-02992-w)
Supplement: Supplementary file 1 — Supplementary Material 1 [file 12866_2023_2992_MOESM1_ESM.docx]

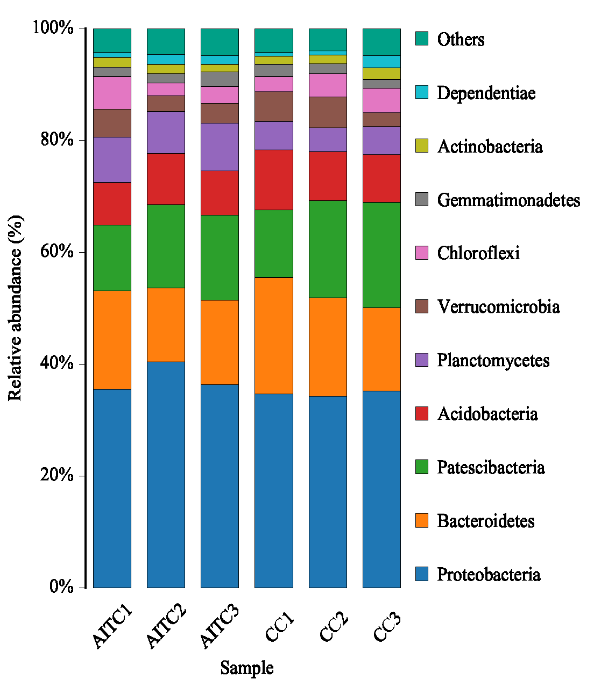

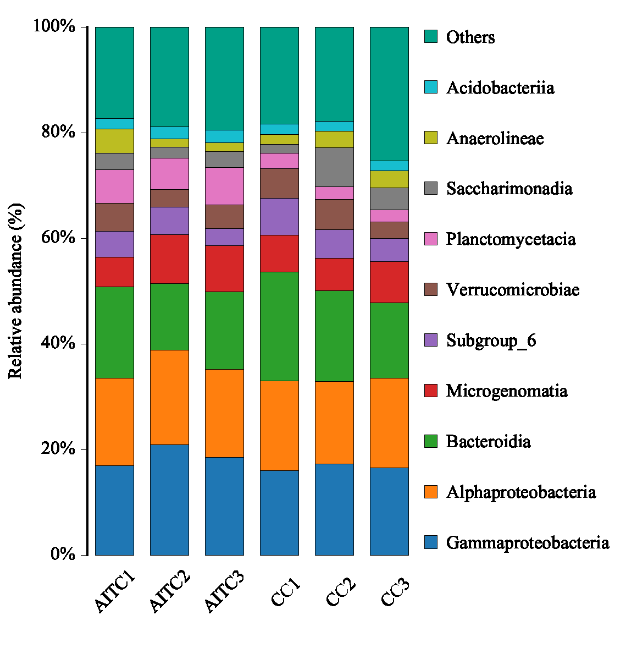

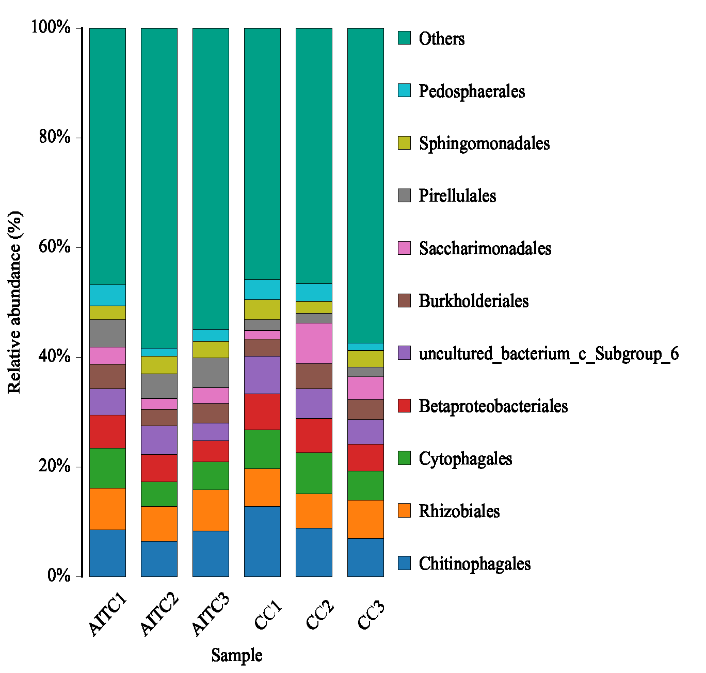

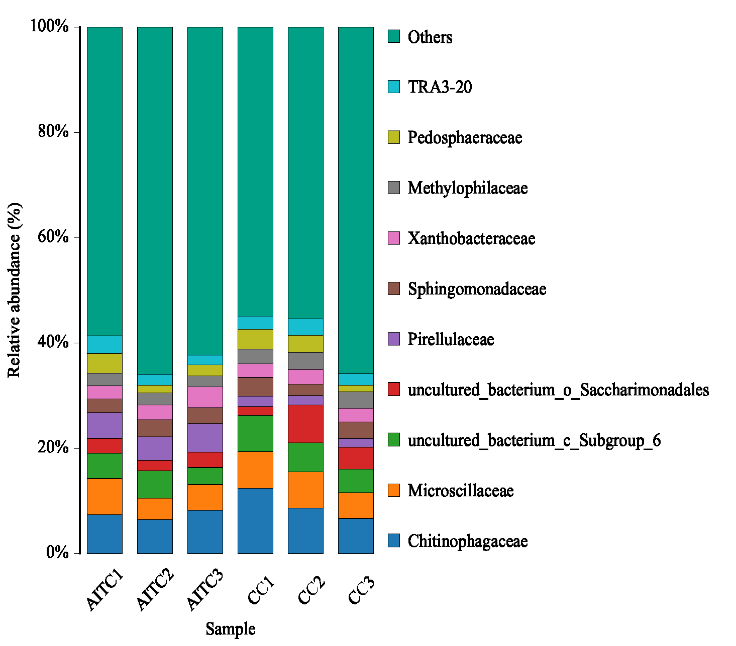


**(a)**

**(b)**

**(c)**

**(d)**

**Supplementary Figure S1:** The top 10 bacterial communities at the level of phylum, class, order, and family, respectively. *n* = 3 for the CC/AITC treatment group. The rest of the top 10 bacterial communities were combined as “others”.
