# Supplementary material for: Effects of allyl isothiocyanate fumigation on medicinal plant root knot disease control, plant survival, and the soil bacterial community
Source: BMC Microbiol. 2023 Sep 30;23:278. doi: 10.1186/s12866-023-02992-w (PMC10542678; doi:10.1186/s12866-023-02992-w)
Supplement: Supplementary file 2 — Supplementary Material 2 [file 12866_2023_2992_MOESM2_ESM.docx]

**Supplementary table S1: Genus-level reductions of relative abundance after treatment of CC soil with AITC (*p* < 0.05)**

| genus | AITC | CC | *p* value |
| --- | --- | --- | --- |
| Povalibacter | 0.00217±0.000188 | 0.0047±0.000435 | 0.00433 |
| Bryobacter | 0.00307±0.00035 | 0.00539±0.000353 | 0.0081 |
| Steroidobacter | 0.000131±0.000131 | 0.000761±0.0000722 | 0.0115 |
| Lacunisphaera | 0.000419±0.000229 | 0.00145±0.000213 | 0.027 |
| Coxiella | 0.000356±0.000182 | 0.00125±0.000212 | 0.0293 |
| Holophaga | 0 | 0.000965±0.00034 | 0.0391 |
| Fodinicola | 0 | 0.000483±0.000181 | 0.0493 |
| uncultured_bacterium_o_SAR324_cladeMarine_group_B | 0 | 0.000899±0.0000652 | 0 |
| uncultured_bacterium_o_Candidatus_Collierbacteria | 0.00385±0.000569 | 0.0126±0.00143 | 0.00367 |
| uncultured_bacterium_o_211ds20 | 0.000131±0.000131 | 0.00111±0.000143 | 0.00567 |
| uncultured_diatom | 0 | 0.000896±0.000245 | 0.0189 |
| uncultured_bacterium_o_Candidatus_Curtissbacteria | 0 | 0.000415±0.000119 | 0.0216 |
| uncultured_bacterium_f_Hyphomicrobiaceae | 0 | 0.000416±0.000123 | 0.0253 |
| uncultured_bacterium_c_OM190 | 0.00148±0.000443 | 0.00448±0.000873 | 0.032 |
| uncultured_bacterium_f_Roseiflexaceae | 0 | 0.000555±0.000186 | 0.0337 |
| uncultured_bacterium_f_CPla-3_termite_group | 0.000288±0.000146 | 0.00124±0.000311 | 0.0428 |
