# Supplementary material for: Effects of allyl isothiocyanate fumigation on medicinal plant root knot disease control, plant survival, and the soil bacterial community
Source: BMC Microbiol. 2023 Sep 30;23:278. doi: 10.1186/s12866-023-02992-w (PMC10542678; doi:10.1186/s12866-023-02992-w)
Supplement: Supplementary file 3 — Supplementary Material 3 [file 12866_2023_2992_MOESM3_ESM.docx]

**Supplementary table S2 Genus-level increases of relative abundance after treatment of CC soil with AITC (*p*<0.05)**

| genus | AITC | CC | *p* value |
| --- | --- | --- | --- |
| Pir4_lineage | 0.0118±0.00133 | 0.00416±0.00127 | 0.0121 |
| Rhodoplanes | 0.00354±0.000324 | 0.00146±0.00044 | 0.0151 |
| Prevotellaceae_UCG-001 | 0.00199±0.000468 | 0.000553±0.000137 | 0.0344 |
| Pirellula | 0.0136±0.00235 | 0.00601±0.00136 | 0.0401 |
| Variovorax | 0.00146±0.000178 | 0.000758±0.000179 | 0.0422 |
| Flavitalea | 0.0154±0.00123 | 0.0109±0.00108 | 0.0425 |
| uncultured_bacterium_f_Pirellulaceae | 0.0239±0.00306 | 0.00802±0.000725 | 0.00533 |
| uncultured_bacterium_o_Candidatus_Woesebacteria | 0.0209±0.00194 | 0.011±0.00151 | 0.0128 |
| uncultured_bacterium_c_JG30-KF-CM66 | 0.000487±0.0000594 | 0.000139±0.0000695 | 0.0155 |
| uncultured_bacterium_o_CCD24 | 0.00995±0.0006 | 0.00712±0.000628 | 0.0276 |
| uncultured_bacterium_o_Microtrichales | 0.00215±0.000429 | 0.000831±0.000125 | 0.035 |
| uncultured_bacterium_o_Babeliales | 0.000644±0.000159 | 0.000139±0.0000695 | 0.0354 |
